# Use of the HR index to predict maximal oxygen uptake during different exercise protocols

**DOI:** 10.1002/phy2.124

**Published:** 2013-10-23

**Authors:** Jeannie M Haller, Patricia C Fehling, David A Barr, Thomas W Storer, Christopher B Cooper, Denise L Smith

**Affiliations:** 1 Department of Health and Exercise Sciences, Skidmore CollegeSaratoga Springs, New York; 2 David Geffen School of Medicine, University of CaliforniaLos Angeles, Los Angeles, California

**Keywords:** Cardiorespiratory fitness, exercise testing, prediction equation, resting heart rate

## Abstract

This study examined the ability of the HR_index_ model to accurately predict maximal oxygen uptake (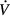
O_2max_) across a variety of incremental exercise protocols. Ten men completed five incremental protocols to volitional exhaustion. Protocols included three treadmill (Bruce, UCLA running, Wellness Fitness Initiative [WFI]), one cycle, and one field (shuttle) test. The HR_index_ prediction equation (METs = 6 × HR_index_ − 5, where HR_index_ = HR_max_/HR_rest_) was used to generate estimates of energy expenditure, which were converted to body mass-specific estimates of 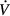
O_2max_. Estimated 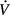
O_2max_ was compared with measured 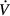
O_2max_. Across all protocols, the HR_index_ model significantly underestimated 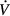
O_2max_ by 5.1 mL·kg^−1^·min^−1^ (95% CI: −7.4, −2.7) and the standard error of the estimate (SEE) was 6.7 mL·kg^−1^·min^−1^. Accuracy of the model was protocol-dependent, with 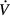
O_2max_ significantly underestimated for the Bruce and WFI protocols but not the UCLA, Cycle, or Shuttle protocols. Although no significant differences in 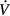
O_2max_ estimates were identified for these three protocols, predictive accuracy among them was not high, with root mean squared errors and SEEs ranging from 7.6 to 10.3 mL·kg^−1^·min^−1^ and from 4.5 to 8.0 mL·kg^−1^·min^−1^, respectively. Correlations between measured and predicted 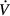
O_2max_ were between 0.27 and 0.53. Individual prediction errors indicated that prediction accuracy varied considerably within protocols and among participants. In conclusion, across various protocols the HR_index_ model significantly underestimated 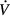
O_2max_ in a group of aerobically fit young men. Estimates generated using the model did not differ from measured 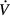
O_2max_ for three of the five protocols studied; nevertheless, some individual prediction errors were large. The lack of precision among estimates may limit the utility of the HR_index_ model; however, further investigation to establish the model's predictive accuracy is warranted.

## Introduction

High cardiorespiratory fitness (CRF) is associated with health benefits, a lower risk of all-cause mortality, (Blair et al. 1989; Kodama et al. 2009; Lee et al. 2011) and a high physical work capacity (Astrand 1956; Balke and Ware 1959). CRF is assessed for diagnostic and prognostic objectives, the evaluation of fitness, the development of exercise prescriptions, and the appraisal of training programs; hence the assessment of CRF is of interest to researchers and clinicians alike. Maximal oxygen uptake (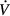
O_2max_) is considered the criterion measure of CRF (American College of Sports Medicine [ACSM] 2006). Direct measurement of 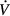
O_2max_, however, requires expensive laboratory equipment, trained personnel, and does not lend itself to testing large numbers of individuals; therefore, 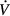
O_2max_ is often estimated indirectly rather than measured.

Estimates of 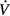
O_2max_ obtained using maximal exercise protocols are typically based on a performance measure such as time or distance covered (Balke and Ware 1959; Cooper 1968; Bruce et al. 1973; Cureton et al. 1995) or in cycle ergometry, peak work rate (Storer et al. 1990). Alternatively, prediction models employing submaximal exercise tests, generally base predictions on the heart rate (HR) response and its well-established linear relationship with oxygen uptake (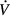
O_2_) over a wide range of exercise intensities (Astrand and Ryhming 1954; Asmussen and Hemmingsen 1958; Margaria et al. 1965). However, it is equally well known that the use of a submaximal HR response to estimate 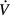
O_2_ involves assumptions that do not always hold true, such as a uniform age-related maximal heart rate (HR_max_) and the linearity of the HR and 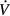
O_2_ relationship (Davies et al. 1984; Shephard 1984).

Recently, Wicks et al. (2011) conducted a retrospective analysis of data extracted from 60 published studies and investigated the relationship between various HR measures and oxygen uptake. The authors concluded that the prediction model employing the ratio of HR during exercise (HR_absolute_) to resting HR (HR_rest_), which was termed the HR_index_, was the preferred model and could be used to predict submaximal and maximal 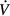
O_2_. Furthermore, the researchers indicated that the HR_index_ method was independent of testing mode (e.g., treadmill, cycle, free-range-activity) and accounted for factors known to contribute to variability in 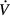
O_2max_, including age, sex, fitness, and body mass.

A prediction model that accurately predicts 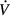
O_2max_ independent of protocol from readily obtained variables would be an attractive tool for field, laboratory, and clinical settings. Wicks et al. (2011) recently reported that their HR_index_ prediction equation could accurately predict 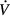
O_2max_. Therefore, the purpose of this exploratory study was to examine the validity of the HR_index_ prediction equation proposed by Wicks et al. (2011) in predicting 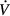
O_2max_ in healthy, active subjects performing a variety of maximal incremental protocols.

## Methods

### Experimental design

This retrospective study utilized data from a parent study that examined the effects of exercise protocol and mode on cardiovascular and metabolic responses to graded exercise testing. These data were further analyzed to investigate the accuracy of the HR_index_ model for predicting 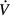
O_2max_. The parent study employed a within-subjects repeated measures design in which participants completed five different incremental exercise tests to volitional exhaustion. Incremental tests were presented in a randomized order and completed on different days. Experimental trials were completed at the same time of day and separated by at least 48 h; all trials were completed within a 14-day period. Participants were instructed to stay well hydrated and to maintain their current diet and exercise patterns throughout the data collection period.

### Participants

Ten healthy, physically active, college-aged men were recruited from the campus community. Individuals were given a detailed account of the study and all participants provided written informed consent prior to the initiation of study procedures. All participants completed a medical history and received a medical evaluation from a health care provider prior to participation in the study. Exclusion criteria included diagnosed cardiorespiratory diseases, use of medication known to alter HR or metabolic rate, or orthopedic problems that interfered with performance of the tests. The study was approved by the college's Institutional Review Board.

### Experimental trial

Participants fasted and abstained from caffeine and nicotine in the 4 h preceding the test and abstained from strenuous exercise and alcohol within 24 h of testing. Hydration status was assessed via urine specific gravity (Schueco Clinical Refractometer 5711-2020; Erma Inc, Tokyo, Japan) to ensure that participants were tested in a euhydrated state (USG ≤ 1.020) (Sawka et al. 2007). Height was measured prior to the first experimental trial using a stadiometer (Seca, Hanover, MD; accuracy ± 0.01 m). Body mass was measured prior to each experimental trial (Befour Inc., Saukville, WI; accuracy ± 0.1 kg). All participants had previous experience completing graded exercise tests to volitional exhaustion; details for each protocol were provided prior to each incremental test. Resting measurements were obtained in a thermoneutral laboratory (21.6 ± 1.2°C; 48.1 ± 6.8% relative humidity) before the incremental test. Participants were outfitted with a portable metabolic measurement system and HR monitor and were instructed to sit quietly for a 10-min period while resting HR and 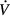
O_2_ data were collected.

Participants then completed one of five incremental protocols: Bruce, UCLA running, Wellness Fitness Initiative (WFI), Shuttle, and Cycle. Protocols were chosen to represent common modes used in clinical and performance exercise tests (running and cycling), different stage durations and workload increments within a mode, an occupationally relevant test (WFI), and a field test (Shuttle). The Bruce, UCLA, and WFI protocols were completed on a motorized treadmill (PPS Med; Woodway USA Inc., Waukesha, WI). The Shuttle run was administered in an indoor gymnasium and the Cycle protocol was performed on an electronically braked cycle ergometer (Velotron; RacerMate Inc., Seattle, WA). During all protocols, verbal encouragement was provided to promote maximal effort. Tests were terminated upon volitional exhaustion of the participant or the participant's inability to maintain the target cadence or speed.

The Bruce protocol (Bruce et al. 1973) included 3-min stages, with the first stage beginning at a gradient of 10% and a speed of 2.7 km·h^−1^. At the end of each stage the gradient increased by 2%; the speed increased to 4.0, 5.5, 6.8, 8.0, and 8.8 km·h^−1^ for the subsequent stages.

The UCLA running protocol consisted of 1-min stages. During the first 3 min, the gradient was held at 0% and the speed increased from 4.8 to 5.5 to 6.0 km·h^−1^. Between minutes 3 and 12, the treadmill gradient was set at 2% and speed was increased by 1.1 or 1.3 km·h^−1^ each minute until the maximum speed of 16.7 km·h^−1^ was reached. At the beginning of minute 12, the gradient was increased by 2% each minute while the treadmill speed remained constant at 16.7 km·h^−1^.

For the WFI (National Fire Protection Association [NFPA] 2006), the gradient was held at 0% and the speed at 4.8 km·h^−1^ for the first 3 min. The speed was then increased to 7.2 km·h^−1^ while the gradient remained at 0%. The treadmill gradient and speed were then alternately increased by 2% and 0.8 km·h^−1^, respectively, at the end of each minute.

The Cycle protocol began at a power output of 60 watts for 3 min. Thereafter, the work rate was increased by 40 watts (Heyward 2010) at the end of every 2-min stage. Target cadence was 60 revolutions per min.

For the Shuttle test (Léger et al. 1988), participants were required to run between two lines located 20 m apart at a set pace that was established using recorded signals. The test started at a speed of 8.5 km·h^−1^ and was increased by 0.5 km·h^−1^ every min. If the participant failed to cover the distance between signal emissions, a warning was given. If the participant failed to cover the distance on two consecutive lengths, the test was terminated.

### Measurements

Oxygen uptake was measured continuously during the testing session using a portable metabolic system (Oxycon Mobile; Care Fusion, Yorba Linda, CA). Before each testing session, ambient temperature and pressure, delay, gas, and volume calibrations were performed. The Oxycon Mobile was worn on the back in a specially designed harness. Expired air was collected with a face mask connected to the flow sensor unit and sampling line of the Oxycon Mobile. Data were transmitted wirelessly to a personal computer. Breath-by-breath data were averaged over 15-sec intervals.

Heart rate was measured continuously throughout the testing session (Zephyr BioHarness BT2, Annapolis, MD). Following testing, HR data were downloaded to a laptop and stored for subsequent analysis. HR data were temporally aligned with 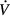
O_2_ data and the second-by-second data were averaged over 15-sec intervals.

During each trial, the participant indicated his rating of perceived exertion (RPE) using the 6–20 Borg Scale (Borg 1982). Incremental tests were considered maximal if two of the following three criteria were met: (1) a plateau in oxygen uptake despite an increase in work rate, (2) respiratory exchange ratio (R) ≥1.1, and (3) a HR within 12 beats of age-predicted maximal HR (HR_max_) (Plowman and Smith 2014).

Resting HR was identified as the lowest HR among the eight 15-sec intervals between minutes 7 and 9 of the rest period. The highest HR among all 15-sec intervals during the incremental test was considered HR_max_. The HR_index_ was calculated using the following equation: HR_index_ = HR_max_/HR_rest_. The HR_index_ was used to predict energy expenditure using the equation proposed by Wicks et al. (2011):


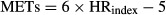
(1)

Predicted energy expenditure in METs was converted to mass-specific 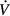
O_2_ using the conversion factor of 3.5 mL·O_2_·kg^−1^·min^−1^ per 1 MET. The conversion factor was selected to correspond to the factor used by Wicks et al. (2011) when converting body mass-specific 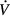
O_2_ to METs in the development of the HR_index_ prediction model. The highest measured 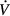
O_2max_ value among all 15-sec intervals during the incremental test was identified as the criterion 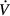
O_2max_.

### Statistical analyses

Data are presented as mean ± SD unless indicated otherwise. A one-way (protocol) analysis of variance with repeated measures was used to detect differences in HR_rest_, HR_max_, HR_index_, and 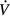
O_2max_ across the protocols. The accuracy of the 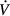
O_2max_ predictions was assessed by computing the bias (mean difference between predicted and measured 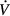
O_2max_) and 95% confidence intervals (CI) for each protocol and overall. If the CI did not include zero, predicted and measured 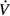
O_2max_ were considered statistically different at an alpha level of 0.05. Precision of the predictions overall and for each protocol were assessed using the root mean squared error (RMSE) and the standard error of the estimate (SEE). The RMSE is the square root of the mean of the squared prediction errors (predicted 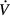
O_2max_ − measured 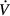
O_2max_) and expresses the total error of the prediction equation, which includes the variation due to the lack of association between two measurements quantified by the SEE (Lohman 1981). A Bland–Altman plot was constructed to depict the overall bias and display any systematic error in the prediction. Pearson product-moment correlation coefficients (*r*) were computed to describe the strength of the linear relationship between measured and predicted 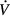
O_2max_. Statistical analyses were performed using the Statistical Package for the Social Sciences (SPSS, Inc., Chicago, IL; software version 19). The level of statistical significance was set at *P* < 0.05.

## Results

Ten young, fit men (age 22 ± 2 year; height 1.76 ± 0.08 m; body mass 78.0 ± 8.5 kg; body mass index 25.2 ± 2.7 kg·m^−2^) completed the study. On average, participants engaged in regular aerobic physical activity for ∼40–45 min on 3 day·week^−1^. All incremental tests were considered maximal based on the attainment of at least two of the three established criteria described in the Methods section.

Heart rate and metabolic data are summarized in Table 1. There were no differences in HR_rest_ (*P* = 0.091) or HR_max_ (*P* = 0.183) among trials. The HR_index_ was significantly lower for the Cycle compared with the Shuttle protocol (*P* = 0.002). No significant differences were detected for other HR_index_ contrasts, however, there was a trend for the HR_index_ to be lower for the Cycle than the UCLA protocol (*P* = 0.058). There was no difference in R among protocols (*P* = 0.053). 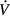
O_2max_ was significantly greater for the Bruce (*P* = 0.004) and WFI (*P* = 0.026) protocols compared with the Cycle protocol.

**Table 1:** Heart rate, metabolic, and perceptual effort among protocols (*N* = 10).

Variable	Bruce	UCLA	WFI	Shuttle	Cycle
HR_rest_ (beats·min^−1^)	62 ± 8	61 ± 7	62 ± 5	59 ± 6	65 ± 6
HR_max_ (beats·min^−1^)	187 ± 9	192 ± 12	192 ± 8	189 ± 11	186 ± 11
HR_index_	3.0 ± 0.4	3.2 ± 0.4	3.1 ± 0.3	3.3 ± 0.4*	2.9 ± 0.3
RPE_peak_	19.1 ± 0.9	19.7 ± 0.5	19.6 ± 0.5	19.3 ± 1.0	19.3 ± 0.7
R_peak_	1.29 ± 0.06	1.27 ± 0.05	1.25 ± 0.08	1.23 ± 0.04	1.27 ± 0.05
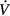 O_2max_ (mL·kg^−1^·min^−1^)	54.9 ± 7.5*	52.2 ± 8.0	54.1 ± 6.8*	52.9 ± 5.0	47.8 ± 8.3

Values are mean ± SD. HR_rest_, resting heart rate; HR_max_, maximum heart rate; HR_index_, HR_max_/HR_rest_; RPE, rating of perceived exertion; R, respiratory exchange ratio; 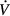
O_2max_, maximal oxygen uptake; WFI, Wellness Fitness Initiative.

* *P* < 0.05 compared with the Cycle protocol for the given variable.

**Figure 1:**
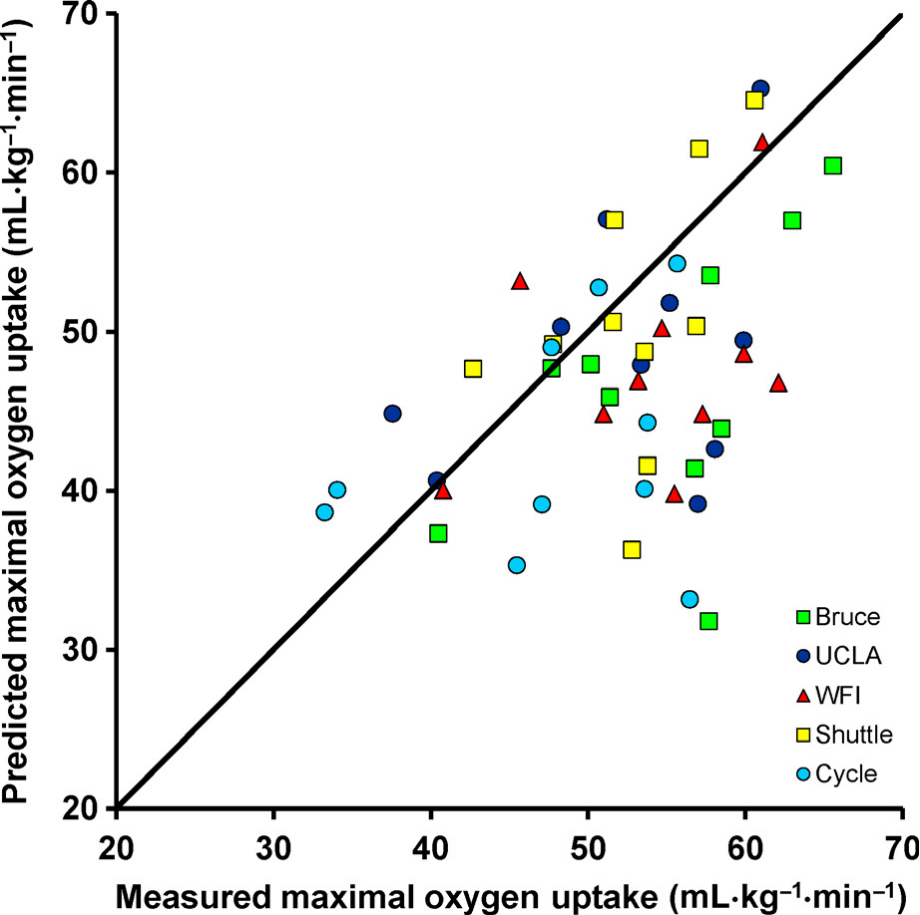
Scatter plot of predicted versus measured 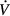
O_2max_ with points identified by protocol. The line of identity denotes perfect agreement.

Figure 1 presents predicted versus measured 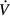
O_2max_ for all trials. Table 2 provides a comparison of measured versus predicted 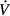
O_2max_, including prediction bias, RMSE, SEE and correlation between measured and predicted 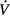
O_2max_. Over all trials, the prediction equation significantly underestimated 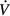
O_2max_ by 5.1 ± 8.3 mL·kg^−1^·min^−1^ when compared with measured 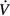
O_2max_ (95% CI = −7.4, −2.7). The negative bias for all protocols indicated a consistent underestimation of 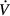
O_2max_ on average by HR_index_ model, whereas a 95% CI that did not span zero for the Bruce and WFI protocols indicated a significant difference between predicted and measured 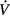
O_2max_. The RMSE was 9.6 mL·kg^−1^·min^−1^ overall and ranged from 7.6 (Shuttle) to 11.2 mL·kg^−1^·min^−1^ (Bruce) among the five protocols. The SEE was lowest for the Shuttle and highest for the Cycle protocol. Low to moderate correlations between measured and predicted 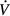
O_2max_ were identified.

**Table 2:** Comparison of measured and predicted maximal oxygen uptake (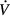
O_2max_).

	Bias (95% CI) (mL·kg^−1^·min^−1^)	RMSE (mL·kg^−1^·min^−1^)	SEE (mL·kg^−1^·min^−1^)	*r*
Bruce	−8.3 (−14.0, −2.6)*	11.2	6.4	0.53
UCLA	−3.3 (−9.7, 3.0)	9.1	7.4	0.38
WFI	−6.4 (−11.8, −1.1)*	9.6	6.3	0.36
Shuttle	−2.1 (−7.7, 3.4)	7.6	4.5	0.45
Cycle	−5.2 (−11.9, 1.6)	10.3	8.0	0.27
Overall	−5.1 (−7.4, −2.7)*	9.6	6.7	0.42

*N* = 50 for Overall and *N* = 10 for all other protocols. WFI, Wellness Fitness Initiative; Bias, predicted 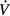
O_2max_ – measured 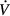
O_2max_; CI, confidence interval; RMSE, root mean squared error; SEE, standard error of the estimate; *r*, Pearson product-moment correlation.

* Predicted 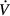
O_2max_ significantly different than measured 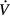
O_2max_.

**Figure 2:**
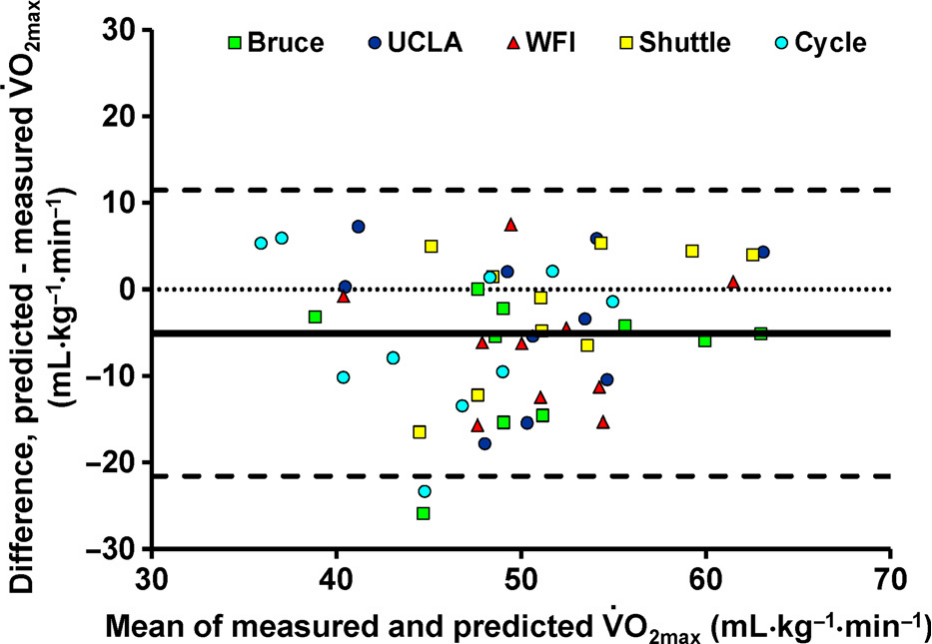
Bland–Altman plot of the prediction errors (predicted 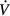
O_2max_ – measured 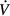
O_2max_). The bias (solid line) and the limits of agreement (dashed lines; mean difference ± 1.96 SD) are displayed.

The Bland–Altman plot (Fig. 2) displays the dispersion of the individual prediction errors and the overall bias and limits of agreement. The wide limits of agreement (11.5, −21.6 mL·kg^−1^·min^−1^) reflect the variability among the prediction errors. Table 3 presents the accuracy of predictions on an individual level, clearly showing that prediction accuracy varies considerably among participants as well as by protocol within participants.

**Table 3:** Individual prediction errors (predicted 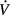
O_2max_ − measured 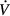
O_2max_) across all trials.

Part #	Protocol				

	Bruce	UCLA	WFI	Shuttle	Cycle
01	−25.9	2.0	−6.2	−4.9	−13.5
02	0.0	7.2	7.5	1.4	5.3
03	−6.0	−10.5	−15.3	−1.0	−9.6
04	−2.3	−5.5	−4.5	−12.3	−10.2
05	−5.5	−3.4	−15.7	5.3	−8.0
06	−4.3	5.8	−6.3	4.4	2.1
07	−15.4	−17.9	−12.5	−16.5	−23.4
08	−3.2	0.2	−0.8	4.9	5.9
09	−5.2	4.3	0.8	3.9	−1.5
10	−14.6	−15.5	−11.3	−6.6	1.3

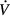
O_2max_, maximal oxygen uptake; Part #, participant number; WFI, Wellness Fitness Initiative; units are mL·kg^−1^·min^−1^.

## Discussion

Across all test protocols, this study found that the HR_index_ prediction model significantly underestimated 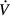
O_2max_ in aerobically fit, college-aged men. Furthermore, prediction accuracy was influenced by the incremental test protocol utilized. 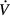
O_2max_ was significantly underpredicted for the Bruce and WFI protocols; however, no significant differences between estimated and measured 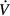
O_2max_ were found for the UCLA running, Shuttle, or Cycle protocols. In addition, accuracy of predictions at the individual level was highly variable for all protocols.

CRF is a measure of interest to many clinicians, researchers, and other practitioners who perform graded exercise testing, and the use of prediction equations to estimate CRF is routinely employed, particularly in large-scale studies. In their development of the HR_index_ model, Wicks et al. (2011) identified published studies that reported measured values for HR_rest_, HR_absolute_, and 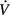
O_2_ and extracted 220 data points that were group averages from 60 eligible exercise studies. The large dataset included diverse populations, different modes of exercise, and both submaximal and maximal data. Prediction models were developed to estimate energy expenditure over the range of 1–14 METs (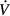
O_2max_ range: 3.5–49.0 mL·kg^−1^·min^−1^) using HR as a predictor, with HR expressed as either HR_absolute_, HR_net_ (HR_absolute_ − HR_rest_) or HR_index_. The researchers concluded that the best fit model employed the HR_index_, which explained 99.1% of the variability in the data. Moreover, subgroup analysis, which included testing device (treadmill, cycle ergometer, other), indicated that the prediction equation was robust. Therefore, a single prediction equation, rather than protocol-specific or sex-specific equations, for the prediction of energy expenditure was proposed by Wicks et al. (2011). These researchers used data points based on group means rather than individual data point to develop the HR_index_ model, and it remains unclear how this might impact model performance. Furthermore, the HR_index_ prediction equation was not cross validated; therefore, subsequent performance of the model was uncertain. To our knowledge, no published studies have examined the accuracy of the HR_index_ prediction equation. In this study, the HR_index_ prediction equation was applied using five protocols that included three exercise types. 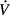
O_2max_ was significantly underestimated overall and a difference in prediction accuracy among protocols was found.

In theory, assuming a uniform HR_rest_, the direct relationship between HR_max_ and 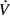
O_2max_ in the HR_index_ model dictates that for estimates of 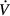
O_2max_ to be the same for two protocols, HR_max_ and 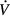
O_2max_ must remain the same or change in a similar way between protocols. A review of several studies that compared cycling versus running or different treadmill protocols revealed that the effect of exercise protocol on 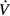
O_2max_ corresponded to the effect of exercise protocol on HR_max_ in some studies (McArdle and Magel 1970; Miyamura et al. 1978; Pannier et al. 1980; Verstappen et al. 1982; Davies et al. 1984; Fernhall and Kohrt 1990) but not others (Hermansen and Saltin 1969; Faulkner et al. 1971; Froelicher et al. 1974; St Clair Gibson et al. 1999). Thus, a precise estimate of 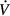
O_2max_ using the HR_index_ model, which includes HR_max_ as a predictor, may be difficult to obtain across different protocols. Results from the present investigation support this point. As shown in Table 1, measured 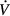
O_2max_ was greater for the Bruce and WFI protocols than the Cycle protocol; however, HR_max_ did not differ among protocols. As the effect of protocol was not the same for HR_max_ and 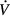
O_2max_, it was not surprising that differences between predicted and measured 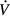
O_2max_ were observed. Additionally, the combined effect of small nonsignificant differences in HR_max_ and HR_rest_ likely led to the significantly lower HR_index_ for the Cycle than the Shuttle protocol. Accordingly, estimated 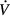
O_2max_ followed the same pattern as the HR_index_, which did not match measured 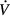
O_2max_.

Wicks et al. (2011) developed the HR_index_ model using group averages, with 5–1909 participants contributing to a data point; therefore, the researchers were unable to establish the prediction error for an individual. Examination of the individual prediction errors in this study (Fig. 2 and Table 3) indicated a wide range of errors within all protocols. Moreover, the prediction errors displayed considerable variability in predictive accuracy among individuals across protocols. Uth et al. (2004) used the ratio of HR_max_ to HR_rest_ (i.e., the HR_index_) to estimate 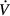
O_2max_, but the researchers derived a prediction equation, which included a proportionality constant and the HR_index_ as factors, based on the Fick equation rather than regressing 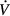
O_2max_ on HR as Wicks et al. (2011) did. In a group of 46 well-trained men (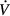
O_2max_ = 60.9 ± 5.5 mL·kg^−1^·min^−1^), Uth et al. (2004) determined the proportionality constant in one subgroup (*n* = 10) and then predicted 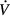
O_2max_ in the second subgroup (*n* = 36). The researchers reported a nonsignificant difference of 0.28 mL·kg^−1^·min^−1^ between measured and predicted 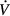
O_2max_ and an SEE of 2.7 mL·kg^−1^·min^−1^, indicating good agreement between measured and predicted 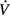
O_2max_ in the group of well-trained men. Validation studies of maximal performance tests based on time or work rate have reported a wide range of predictive accuracies, nevertheless, the predictive accuracy of commonly used equations (Foster et al. 1984; Storer et al. 1990; American College of Sports Medicine 2006; Heyward 2010) is higher than the accuracy noted for the HR_index_ in this study. Storer et al. (1990) reported an SEE of 2.57 mL·kg^−1^·min^−1^ for the prediction of 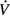
O_2max_ from body mass, work rate, and age in cycle ergometry using a sex-specific equation for men. A generalized prediction equation frequently used with the Bruce protocol has an SEE of 3.35 mL·kg^−1^·min^−1^ (Foster et al. 1984), which is considerably lower than the 6.4 mL·kg^−1^·min^−1^ identified in this study. In fact, this regression equation for the Bruce protocol, which uses test time to predict 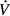
O_2max_, produced accurate estimates of 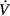
O_2max_ when applied to the data collected in this investigation (prediction bias = −2.6 ± 3.2 mL·kg^−1^·min^−1^; SEE = 3.3 mL·kg^−1^·min^−1^; *r* = 0.90).

There are several possible explanations for the underestimation of 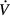
O_2max_ by the HR_index_ model and the high variability in the estimates of 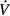
O_2max_. One possible reason is that the participants in this study had a higher CRF on average than those in the model development study. For 72% of the incremental tests in this study, the 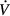
O_2max_ attained was higher than the 49 mL·kg^−1^·min^−1^ upper limit identified in development of the HR_index_ prediction model. It then follows that HR_index_ values in this study likely exceeded or were near the upper limit of those included as predictors by Wicks et al. (2011). However, Wicks et al. (2011) reported that the HR_index_ model accounted for fitness. Our results indicate that the HR_index_ model did not accurately predict 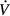
O_2max_ in young, fit men. Furthermore, this study found relatively large prediction errors (>7 mL·kg^−1^·min^−1^) in ∼30% of the trials where 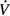
O_2max_ did not exceed 49 mL·kg^−1^·min^−1^, indicating that individual prediction errors may be substantial even within the scope of the prediction model.

Both HR_max_ and HR_rest_ are predictors in the HR_index_ model; therefore, a difference in the measurement of these predictors could affect prediction accuracy. Only 20% of the studies included in the development of the HR_index_ model indicated the methods used to obtain HR_rest_. Moreover, among those studies providing details, procedures were vastly different, with rest periods between 2 and 90 min and inconsistency in the position of the participants (seated or supine). In this study, the method for obtaining HR_rest_ was guided by the technology used and the potential application of the HR_index_ method in a typical cardiopulmonary exercise test setting where prediction of 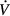
O_2max_ is the objective. Therefore, the measurement was obtained with the participant in a seated rather than supine position. Additionally, the use of the lowest 15-sec sampling interval was easily achieved using a HR monitor, and helped to ensure the obtainment of a HR_rest_ value in a field study. The rest period of 7 min prior to obtaining the measurement was consistent with methods described for obtaining HR_rest_ (American College of Sports Medicine 2008; Heyward 2010). In part, the underestimation of 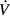
O_2max_ may be a consequence of the seated HR_rest_ measurement, but this is not possible to ascertain because the methods for obtaining HR_rest_ were unknown or inconsistent in the studies used to develop the HR_index_ model. Wicks et al. (2011) justifiably indicate that the measurement of HR_rest_ needs to be standardized for the accurate prediction of the HR_index_ and 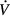
O_2_. Notwithstanding standardization of the HR_rest_ measurement, accuracy of individual level predictions using the HR_index_ model may be variable. Lee et al. (2010) used the HR_index_ to predict MET values of various submaximal activities in persons with paraplegia, and the group reported a substantially lower (∼23%) absolute error percentage when using prediction equations developed for each individual (individual calibration) compared with a single regression equation developed for all participants (group calibration) despite the use of a standardized procedure to determine HR_rest_. Previous research (Andrews 1971; Hiilloskorpi et al. 2003) suggests that the incorporation of HR_rest_ in the predictor reduces but does not eliminate the interindividual differences when using HR to predict energy expenditure.

The accuracy of 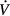
O_2max_ estimates could have been influenced by differences in resting metabolic rate and the use of the standard conversion factor to convert METs to 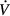
O_2_. This study used the established conversion factor of 3.5 mL·O_2_·kg^−1^·min^−1^ per 1 MET, which is the method used in the development of the HR_index_ model. Although this conversion factor is routinely used, research has shown that this value does not always equate to resting 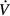
O_2_ (Byrne et al. 2005) and hence this may be a source of error within the HR_index_ model.

This study involved five incremental exercise tests conducted within a 14-day interval. Thus, day to day variability in measurements of HR_max_, HR_rest_, and 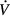
O_2max_ may have contributed to the variability in the predictions between exercise protocols. Day to day variability in HR_max_ is ∼2–4 beats·min^−1^ (Achten and Jeukendrup 2003). The standard deviation of HR_rest_ (5–8 beats·min^−1^) in this study suggested considerable variability in this measure. Procedures had been implemented to limit the effect of factors that influence heart rate, but uncontrollable factors, such as stress level or sleep quality, may have contributed to within-participant variability. For repeated measurements of 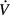
O_2max_, studies have reported a coefficient of variation of 4–5% (Katch et al. 1982; Howley et al. 1995) and a reliability coefficient of 0.95 (Taylor et al. 1955). Thus, trial to trial variability in physiological measures, notably HR_rest_, could account for some variability in predictions.

The retrospective nature of the study resulted in several inherent limitations, namely the dataset was small and obtained on a homogeneous group, limiting statistical power and generalizability of findings. Protocols employed included different exercise types; however, the use of only protocols with established prediction equations would have permitted comparisons between the HR_index_ and protocol-specific equations. Additional research with larger, more heterogeneous groups is needed to further evaluate the performance of the HR_index_ prediction model.

## Conclusions

The use of a simple predictor, such as a HR_index_, to predict 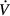
O_2max_ across different exercise protocols is an attractive possibility, and a recently published article (Wicks et al. 2011), which retrospectively analyzed a large number of published studies suggested that this may be possible. However, this study found that the HR_index_ prediction model significantly underestimated 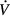
O_2max_ in young, fit men across different protocols. Furthermore, the incremental test protocol influenced prediction accuracy, with predicted 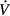
O_2max_ differing significantly from measured 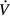
O_2max_ for the Bruce and WFI protocols but not the UCLA running, Shuttle or Cycle protocols. Despite the fact that no significant differences between measured and predicted 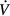
O_2max_ were found for three of the five protocols studied, examination of the individual data revealed large prediction errors among all protocols. Additionally, prediction accuracy across protocols varied considerably within individuals. Therefore, our results suggest caution is warranted when applying the HR_index_ prediction equation to estimate 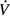
O_2max_ in young, fit men. The findings from this study are consistent with the view that prediction models often provide valid estimates on a group level, but the accuracy of individual estimates vary considerably.

## References

[b1] Achten J, Jeukendrup AE (2003). Heart rate monitoring: applications and limitations. Sports Med.

[b2] American College of Sports Medicine (2006). ACSM's guidelines for exercise testing and prescription.

[b3] American College of Sports Medicine (2008). ACSM's health-related physical fitness assessment manual.

[b42] Andrews RB (1971). Net heart rate as a substitute for respiratory calorimetry. Am. J. Clin. Nutr.

[b4] Asmussen E, Hemmingsen I (1958). Determination of maximum working capacity at different ages in work with the legs or with the arms. Scand. J. Clin. Lab. Invest.

[b5] Astrand PO (1956). Human physical fitness with special reference to sex and age. Physiol. Rev.

[b6] Astrand PO, Ryhming I (1954). A nomogram for calculation of aerobic capacity (physical fitness) from pulse rate during sub-maximal work. J. Appl. Physiol.

[b7] Balke B, Ware RW (1959). An experimental study of physical fitness of air force personnel. US Armed Forces Med. J.

[b8] Blair S, Kohl H, Paffenbarger R, Clark D, Cooper K, Gibbons L (1989). Physical fitness and all-cause mortality: a prospective study of healthy men and women. JAMA.

[b9] Borg G (1982). Psychophysical bases of perceived exertion. Med. Sci. Sports Exerc.

[b10] Bruce RA, Kusumi F, Hosmer D (1973). Maximal oxygen intake and nomographic assessment of functional aerobic impairment in cardiovascular disease. Am. Heart J.

[b11] Byrne NM, Hills AP, Hunter GR, Weinsier RL, Schutz Y (2005). Metabolic equivalent: one size does not fit all. J. Appl. Physiol.

[b12] Cooper KH (1968). A means of assessing maximal oxygen intake. Correlation between field and treadmill testing. JAMA.

[b13] Cureton KJ, Sloniger MA, O'bannon JP, Black DM, Mccormack WP (1995). A generalized equation for prediction of VO_2peak_ from 1-mile run/walk performance. Med. Sci. Sports Exerc.

[b14] Davies B, Daggett A, Jakeman P, Mulhall J (1984). Maximum oxygen uptake utilising different treadmill protocols. Br. J. Sports Med.

[b15] Faulkner JA, Roberts DE, Elk RL, Conway J (1971). Cardiovascular responses to submaximum and maximum effort cycling and running. J. Appl. Physiol.

[b16] Fernhall B, Kohrt W (1990). The effect of training specificity on maximal and submaximal physiological responses to treadmill and cycle ergometry. J. Sports Med. Phys. Fitness.

[b17] Foster C, Jackson AS, Pollock ML, Taylor MM, Hare J, Sennett SM (1984). Generalized equations for predicting functional capacity from treadmill performance. Am. Heart J.

[b18] Froelicher VF, Brammell H, Davis G, Noguera I, Stewart A, Lancaster MC (1974). A comparison of three maximal treadmill exercise protocols. J. Appl. Physiol.

[b19] Hermansen L, Saltin B (1969). Oxygen uptake during maximal treadmill and bicycle exercise. J. Appl. Physiol.

[b20] Heyward VH (2010). Advanced fitness assessment and exercise prescription.

[b43] Hiilloskorpi HK, Pasanen ME, Fogelholm MG, Laukkanen RM, Manttari AT (2003). Use of heart rate to predict energy expenditure from low to high activity levels. Int. J. Sports Med.

[b21] Howley ET, Bassett DR, Welch HG (1995). Criteria for maximal oxygen uptake: review and commentary. Med. Sci. Sports Exerc.

[b22] Katch VL, Sady SS, Freedson P (1982). Biological variability in maximum aerobic power. Med. Sci. Sports Exerc.

[b23] Kodama S, Saito K, Tanaka S, Maki M, Yachi Y, Asumi M (2009). Cardiorespiratory fitness as a quantitative predictor of all-cause mortality and cardiovascular events in healthy men and women: a meta-analysis. JAMA.

[b24] Lee M, Weimo Z, Hedrick B, Fernhall B (2010). Estimating MET values using the ratio of HR for persons with paraplegia. Med. Sci. Sports Exerc.

[b25] Lee DC, Sui X, Ortega FB, Kim YS, Church TS, Winett RA (2011). Comparisons of leisure-time physical activity and cardiorespiratory fitness as predictors of all-cause mortality in men and women. Br. J. Sports Med.

[b26] Léger LA, Mercier D, Gadoury C, Lambert J (1988). The multistage 20 metre shuttle run test for aerobic fitness. J. Sports Sci.

[b27] Lohman TG (1981). Skinfolds and body density and their relation to body fatness: a review. Hum. Biol.

[b28] Margaria R, Aghemo P, Rovelli E (1965). Indirect determination of maximal O_2_ consumption in man. J. Appl. Physiol.

[b29] McArdle WD, Magel JR (1970). Physical work capacity and maximum oxygen uptake in treadmill and bicycle exercise. Med. Sci. Sports.

[b30] Miyamura M, Kitamura K, Yamada A, Matsui H (1978). Cardiorespiratory responses to maximal treadmill and bicycle exercise in trained and untrained subjects. J. Sports Med. Phys. Fitness.

[b31] National Fire Protection Association (2006). NFPA 1582. Standard on comprehensive occupational medical program for fire departments.

[b32] Pannier JL, Vrijens J, Van Cauter C (1980). Cardiorespiratory response to treadmill and bicycle exercise in runners. Eur. J. Appl. Physiol.

[b33] Plowman SA, Smith DL (2014). Exercise physiology for health, fitness, and performance.

[b34] Sawka MN, Burke LM, Eichner ER, Maughan RJ, Montain SJ, Stachenfeld NS (2007). American College of Sports Medicine position stand. Exercise and fluid replacement. Med. Sci. Sports Exerc.

[b35] Shephard RJ (1984). Tests of maximum oxygen intake. A critical review. Sports Med.

[b36] St Clair Gibson A, Lambert MI, Hawley JA, Broomhead SA, Noakes TD (1999). Measurement of maximal oxygen uptake from two different laboratory protocols in runners and squash players. Med. Sci. Sports Exerc.

[b37] Storer TW, Davis JA, Caiozzo VJ (1990). Accurate prediction of VO_2max_ in cycle ergometry. Med. Sci. Sports Exerc.

[b38] Taylor HL, Buskirk E, Henschel A (1955). Maximal oxygen intake as an objective measure of cardio-respiratory performance. J. Appl. Physiol.

[b39] Uth N, Sørensen H, Overgaard K, Pedersen PK (2004). Estimation of VO_2max_ from the ratio between HRmax and HRrest–the heart rate ratio method. Eur. J. Appl. Physiol.

[b40] Verstappen FT, Huppertz RM, Snoeckx LH (1982). Effect of training specificity on maximal treadmill and bicycle ergometer exercise. Int. J. Sports Med.

[b41] Wicks JR, Oldridge NB, Nielsen LK, Vickers CE (2011). HR index-a simple method for the prediction of oxygen uptake. Med. Sci. Sports Exerc.

